# Efficacy of major ozone autohemotherapy in patients with post-COVID syndrome

**DOI:** 10.3389/fmed.2026.1720578

**Published:** 2026-02-13

**Authors:** Ozlem Kuculmez

**Affiliations:** Department of Physical Medicine and Rehabilitation, Baskent University Alanya Hospital, Antalya, Türkiye

**Keywords:** complementary therapies, coronavirus, fatigue, musculoskeletal diseases, ozone, pain

## Abstract

**Background and objectives:**

It has been suggested that medical ozone treatment may be used for post-COVID syndrome, but there is still a lack of evidence. The aim of the study is to investigate the efficacy of medical ozone treatment in fatigue, anxiety, depression, quality of life, and quality of sleep in patients diagnosed with post-COVID syndrome.

**Materials and methods:**

The study was designed as a retrospective medical records review study. It was conducted at Baskent University in patients who had been diagnosed with post-COVID syndrome and accepted 10 sessions of major ozone autohemotherapy. Demographic data, FACIT Fatigue Scale, Beck Depression Inventory, Beck Anxiety Inventory, Pittsburgh Sleep Quality Index, and Short Form−36 scores were obtained from the hospital database. The scores before and after treatment were compared statistically. *p* < 0.05 was revealed as statistically significant.

**Results:**

A total of 40 patients were analyzed. Thirty two of them were female, and eight of them were male. It was determined that both Beck Anxiety Inventory and Beck Depression Inventory scores were decreased after ozone application (*p* < 0.05). Statistically significant decreases in FACIT scores were obtained after 10 sessions of major blood ozonation (*p* < 0.05). Additionally, improvement in all subscores of Short Form-36 was determined after the medical ozone treatment (*p* < 0.05).

**Conclusion:**

The patients suffering from post-COVID syndrome may benefit from major blood ozonation. The treatment may be one of the complementary treatments after COVID-19 infection.

## 1 Introduction

Post-COVID syndrome refers to a set of symptoms (such as fatigue, headaches, shortness of breath, cognitive difficulties, depression, brain fog, skin rashes, and digestive issues) that typically emerge within 12 weeks after a coronavirus disease 2019 (COVID-19) infection and cannot be attributed to any other cause (1). Research indicates that these symptoms may persist in 32.6–87% of hospitalized patients, while the prevalence in non-hospitalized individuals ranges between 30% and 37% (2). Fatigue was the most commonly reported symptom in the post-COVID phase, affecting 64% of patients, followed by arthralgia, which was present in 24.3% of cases (3). These symptoms have been found to negatively impact patients’ quality of life (4). Also, another clinical problem that was reported after COVID infection and decreased the patients’ quality of life was atypical chronic pain syndrome (5).

Although pharmacological treatments play a significant role in managing symptoms during the acute infection phase, there are very few treatment options available for the post-COVID period (6). During this period, exercise and rehabilitation programs, vitamin supplements, and major blood ozonation (MBO) may be preferred (7).

MBO is a kind of integrative approach in which oxigen-ozone mixture (a combination of 3%–5% O_3_ and 97%–95% O_2_) produced by ozone generators has been used (8). The treatment has been used in supportive treatment of diseases such as fibromyalgia, rheumatic disease, chronic fatigue syndrome, and infectious diseases. It has been suggested that MBO may be used both for recovery during COVID infection and in patients who develop post-COVID syndrome after infection. Although the number of studies on the subject increases day by day, the level of scientific evidence is still limited (9, 10). The aim of the study is to investigate the efficacy of major MBO in fatigue, anxiety, depression, quality of life, and quality of sleep in patients diagnosed with post-COVID syndrome.

## 2 Materials and methods

The research was structured as a review of medical records. The approval from the Baskent University Clinical Research Ethics Committee (protocol code: KA23/32, approval no: E-94603339-604.01.02-200104, date of approval 24.01.2023) was obtained, and the study was conducted following the principles of the Declaration of Helsinki. Prior to undergoing MBO, all patients provided their consent. The study took place at Başkent University Alanya Hospital, focusing on individuals affected by post-COVID syndrome. The sample size was determined using the G Power program, with a medium effect size, a significance level of 0.05, and a confidence interval of 0.95. According to this calculation, the minimum required sample size was 40 (11).

The study included patients experiencing musculoskeletal symptoms such as fatigue, joint pain, weakness, and arthralgia, who had previously been diagnosed with COVID-19 (confirmed by a polymerase chain reaction test) and had symptoms lasting for at least 12 weeks. The patients who attended the outpatient clinic and agreed to undergo 10 sessions of MBO for post-COVID syndrome were included in the study. Patients with other systemic diseases that could influence the outcomes or had contraindications for MBO were excluded from the study. Additionally, patients who declined the treatment or did not attend the MBO sessions regularly were also excluded.

Ozone-resistant vacuum glass citrated bottles (PPS citrated bottles containing 12 mL of 3.13% sodium citrate for 150 mL blood), serum sets (PPS serum sets), and injectors (Medivare 50–60 mL injectors) were utilized for the MBO protocol. The treatments were carried out using the Turkozone Blue S medical ozone generator (Ozone Health Services Co. Ltd., Istanbul, Turkey) (CE 1984), which is approved by the Ministry of National Health. A total of 100 cc of blood was collected from patients into the citrated bottles using ozone-resistant serum sets. 100 mL of O₃-O₂ mixture gas was then injected into the bottles with 50 mL ozone injectors over a 5-min period. Afterward, the bottles were inverted, and the ozonated blood was reinfused intravenously into the patients within 15 min using the same serum sets. All materials used were sterile and disposable, and antiseptic conditions were maintained throughout the process. In the first session, a 20 μg/mL concentration was administered (100 mL of blood and 100 mL of O₃-O₂ mixture gas), followed by 25 μg/mL in the second session and 30 μg/mL between the third and tenth sessions. This dosing protocol followed the recommendation to begin treatment with a low dose and gradually increase it. A medium-high dose range was chosen for the patients. MOT was administered 2–3 times per week, totaling 10 sessions, in line with recommendations from the literature (12).

Demographic information, COVID test results, previous diagnoses, medications, MOT session-dose details, outpatient clinic follow-up data, and patient medical histories were retrieved from the hospital database. The scores for the FACIT Fatigue Scale, Beck Depression Inventory, Beck Anxiety Inventory, Pittsburgh Sleep Quality Index, and Short Form-36, which were recorded before and after the 10 sessions of MBO, were also obtained from the hospital system, and the scores obtained before and after treatment were statistically compared.

The FACIT Fatigue Scale, which was validated by Cinar et al., consists of 13 questions that are scored between 0 and 4 points. A maximum of 52 points may be obtained, and a higher score means a higher level of fatigue (13). The Beck Depression Inventory consists of 21 questions. The scale was validated by Kapci et al. (14). A maximum of 63 points may be obtained. Scores between 0 and 16 revealed a mild depressive mood, 17 and 29 a moderate depressive mood, and 30 and 63 a severe depressive mood. The Beck Anxiety Scale, which was validated by Gümüs et al. (15) consists of 21 questions. In this scale, a maximum of 63 points may be obtained. Scores between 8 and 15 revealed a mild anxiety, 16 and 25 a moderate anxiety, and 26 and 63 revealed a severe anxiety mood.

The Pittsburgh Sleep Quality Index was developed by Buysse et al. to evaluate sleep quality in psychiatric and clinical studies (16). The Turkish validity and reliability of the scale was conducted by Ağargün et al. (17) The Pittsburgh Sleep Quality Index, consisting of 18 questions and 7 components, was developed to evaluate sleep disorders and sleep quality in the last month. These components include subjective sleep quality, sleep latency, sleep duration, habitual sleep efficiency, sleep disturbance, sleep medication use, and daytime dysfunction. Scores more than 5 revealed poor sleep quality.

The Short Form-36 (SF-36) is a widely used scale to assess quality of life in healthcare research, and it has been validated in Turkish by Demiral et al. (18). It includes straightforward questions across nine different subscales, such as physical functioning (PF), role limitations caused by physical health (PH), role limitations due to emotional issues (EP), fatigue (F), emotional well-being (EW), social functioning (SF), pain (P), general health (GH), and changes in health (HC). Higher scores on all SF-36 subscales suggest a better quality of life, while lower scores indicate a decline in quality of life.

## 3 Statistical analysis

Data were analyzed with the SPSS program (IBM Corp., Chicago, Illinois, USA 25.00), and all results were interpreted at a significance level of 0.05 and a confidence interval of 95%. Normally distributed values were defined as mean ± SD, non-normally distributed values were defined as median (min-max), and categorical variables were defined as frequency and percentage. A normality test was performed with the Shapiro–Wilk test. Because a normal distribution could not be found for FACIT Fatigue Scale, Beck Depression Scale, Beck Anxiety Scale, Pittsburgh Sleep Quality Index, and Short Form-36 score values, the Wilcoxon signed-rank test was used to compare scores before and after treatment. *p* < 0.05 was revealed as statistically significant.

## 4 Results

A total of 45 patients were enrolled. Due to missing data, five patients were excluded from the study, and a total of 40 patients were evaluated. 80% of the patients were female (*n* = 32), and 20% were male (*n* = 8). The mean age was 55.50 ± 14.92. It was determined that 85% (*n* = 24) of the patients were vaccinated. The most frequent symptom during COVID-19 infection was fever and neck pain, with a rate of 60% (*n* = 24). Although there was 10% (*n* = 4) mild and 10% (*n* = 4) fair lung involvement, no severe lung involvement was detected in these patients. The frequency of symptoms during the COVID-19 infection in the patients diagnosed with post-COVID syndrome was given in Table 1.

**Table 1:** Symptoms during COVID-19 infection.

Symptom	Frequency *n* (%)
Fever	*n* = 24 (60%)
Neck pain	*n* = 24 (60%)
Back pain	*n* = 20 (50%)
Cough	*n* = 18 (45%)
Loss of taste	*n* = 16 (40%)
Sweating	*n* = 14 (35%)
Loss of smell	*n* = 14 (35%)
Dyspnea	*n* = 12 (30%)
Sore throat	*n* = 12 (30%)
Arthralgia	*n* = 10 (25%)
Lung involvement	Mild *n* = 4 (10%), Fair *n* = 4 (10%), Severe *n* = 0 (0%)

The most common symptom during outpatient clinic admission in the post-COVID period was found to be fatigue, with a rate of 90% (*n* = 36). It was determined that this was followed by arthralgia, brain fog, and muscle weakness, respectively. The frequency of symptoms during the post-COVID period was given in Table 2.

**Table 2:** Symptoms during post-COVID syndrome.

Symptom	Frequency *n* (%)
Fatigue	*n* = 36 (90%)
Arthralgia	*n* = 12 (30%)
Fibrofog	*n* = 4 (10%)
Muscle weakness	*n* = 2 (5%)

It was determined that these symptoms continued for more than 12 weeks after the COVID infection, and there was no other reason to explain it. This clinical situation was evaluated as post-COVID syndrome, and 10 sessions of MBO were applied. The patients were evaluated with the FACIT Fatigue Scale, Beck Depression Inventory, Beck Anxiety Inventory, Pittsburgh Sleep Quality Index, and Short Form-36 before and after 10 sessions of ozone therapy, and the scores were documented in the hospital database. A statistical comparison of scores before and after treatment revealed a significant reduction in FACIT Fatigue Scale scores following treatment (*p* = 0.001), indicating an improvement in fatigue levels. Furthermore, an enhanced mood was observed post-treatment, as evidenced by a decrease in both depression and anxiety scores after 10 sessions of MBO. Notably, there were significant improvements in the Beck Depression Inventory (*p* = 0.001) and Beck Anxiety Inventory scores (*p* = 0.002). Given the prevalence of sleep disturbances in the post-COVID period, patients were assessed using the Pittsburgh Sleep Quality Index and its subcomponents before and after treatment. Results demonstrated a statistically significant improvement in global Pittsburgh Sleep Quality Index scores following MOT (*p* = 0.016), suggesting that this treatment may contribute to better sleep quality. The change in FACIT fatigue scale, Beck Depression Inventory, Beck Anxiety Inventory, and global Pittsburgh Sleep Quality Index scores before and after treatment is shown in Table 3.

**Table 3:** Change in functional assessment of chronic illness therapy, Beck anxiety inventory, Beck depression inventory before and after ozone application.

*n* = 40		Median (min-max)	*Z*	*p*-value
FACIT^a^	Pretreatment	31.0 (0–51)	−3.625	0.001*
	Posttreatment	9.5 (0–31)		
Beck anxiety score^a^	Pretreatment	7.5 (0–52)	−3.433	0.001*
	Posttreatment	4.0 (0–15)		
Beck depression score^a^	Pretreatment	5.5 (0–49)	−3.066	0.002*
	Posttreatment	1.0 (0–12)		
Global PSQI^a^	Pretreatment	15 (8–20)	−2.409	0.016*
	Posttreatment	13 (5–20)		

a Wilcoxon signed-rank test *p < 0.05 statistically significant.

FACIT, Functional assessment of chronic illness treatment; PSQI, Pittsburgh Sleep Quality Index.

When the Pittsburgh Sleep Quality Index subscores were evaluated, it was found that there was not any significant change in sleep latency (*p* = 0.059) and sleep efficiency (*p* = 1.000). But there were significantly better scores in subjective sleep quality (*p* = 0.011), sleep duration (*p* = 0.008), and sleep disturbance (*p* = 0.034). There was no change in the use of sleep medication after the treatment (*p* = 0.066), but a significant improvement was detected in daytime dysfunction (*p* = 0.039). The change in the global Pittsburgh Sleep Quality Index score and its subscores before and after treatment is shown in Table 4.

**Table 4:** Change in Pittsburgh sleep quality index and its subscores before and after ozone application.

*n* = 40		Median (min–max)	*Z*	*p*-value
Subjective sleep quality^a^	Pretreatment	2 (0–3)	−2.530	0.011*
	Posttreatment	1 (0–3)		
Sleep latency^a^	Pretreatment	3 (0–5)	−1.890	0.059
	Posttreatment	3 (0–5)		
Sleep duration^a^	Pretreatment	7 (3–10)	−2.646	0.008*
	Posttreatment	7 (3–10)		
Sleep efficiency^a^	Pretreatment	0 (0–3)	0.001	1.000
	Posttreatment	0 (0–3)		
Sleep disturbance^a^	Pretreatment	1 (0–3)	−2.121	0.034*
	Posttreatment	1 (0–2)		
Use of sleep medication^a^	Pretreatment	0 (0–3)	−1.841	0.066
	Posttreatment	0 (0–3)		
Day time dysfunction^a^	Pretreatment	1 (0–3)	−2.069	0.039*
	Posttreatment	0 (0–3)		
Global PSQI^a^	Pretreatment	15 (8–20)	−2.409	0.016*
	Posttreatment	13 (5–20)		

a Wilcoxon signed-rank test *p < 0.05 statistically significant.

PSQI, Pitsburg Sleep Quality Index. Bold values indicate statistically significant *p* < 0.05.

When Short Form-36 and subscores were observed, it was found that there was no significant change in only the emotional well-being parameter after the treatment (*p* = 0.394). There were significantly better results in physical functioning (*p* = 0.005), role limitations caused by physical health (*p* = 0.007), role limitations due to emotional issues (*p* = 0.010), fatigue (*p* = 0.043), social functioning (*p* = 0.007), pain (*p* = 0.002), general health (*p* = 0.013), and changes in health (*p* = 0.004). These findings suggest that MOT may enhance the overall quality of life for patients suffering from Post-COVID syndrome. The change in Short Form-36 and its subscores before and after treatment is shown in Table 5.

**Table 5:** Change in Short Form-36 subscores before and after ozone application.

*n* = 40		Median (min-max)	*Z*	*p*-value
Physical functioning^a^	Pretreatment	62 (0–100)	−2.814	0.005*
	Posttreatment	95 (60–100)		
PH^a^	Pretreatment	37 (20–80)	−2.694	0.007*
	Posttreatment	100 (0–100)		
EP^a^	Pretreatment	33 (12–80)	−2.588	0.010*
	Posttreatment	100 (20–100)		
Energy/fatigue^a^	Pretreatment	50 (12.75–75)	−2.023	0.043*
	Posttreatment	50 (20–90)		
Emotinal well-being^a^	Pretreatment	56 (0–100)	−0.852	0.394
	Posttreatment	60 (0–77.5)		
Social functioning^a^	Pretreatment	50 (12.5–75)	−2.696	0.007*
	Posttreatment	62.5 (25–100)		
Pain^a^	Pretreatment	35 (0–77.5)	−3.070	0.002*
	Posttreatment	77.5 (12.5–90)		
General health^a^	Pretreatment	45 (5–70)	−2.487	0.013*
	Posttreatment	50 (35–90)		
Health change^a^	Pretreatment	25 (25–50)	−2.845	0.004*
	Posttreatment	62.5 (25–75)		

a Wilcoxon signed-rank test *p < 0.05 statistically significant.

PH: Role of limitations due to physical health; EP: Role of limitations due to emotional problems. Bold values indicate statistically significant *p* < 0.05.

## 5 Discussion

In the current study, it was found that most patients with post-COVID syndrome were women, and the average age was 55.50 ± 14.92. It was determined that these patients did not have severe lung involvement, and the most frequent symptom that was expressed in this period was fatigue. After 10 sessions MBO, significantly better depression and anxiety scores were obtained. It was determined that fatigue level significantly decreased after the therapy. Also, significant improvement was observed in Pittsburg Sleep Quality Index parameters except sleep latency, sleep efficiency, and use of sleep medication. Additionally, Short Form-36 scores, except for emotional well-being, were improved after the therapy.

Post-COVID syndrome is characterized as a condition that affects individuals with a history of probable or confirmed COVID-19 infection, typically emerging 3 months after the onset of the illness, with symptoms persisting for at least 2 months and not attributable to any other diagnosis. The term “post-COVID” was introduced because some patients experienced ongoing symptoms after the acute phase of infection (19). This syndrome includes common issues like fatigue, shortness of breath, and cognitive impairment but also involves other symptoms, generally affecting daily activities. These symptoms may either appear for the first time after recovering from the initial COVID-19 infection or continue from the initial illness. Symptoms can also vary in severity or recur over time (20). Several studies have reported prevalence rates ranging from 32% to 83% (19–21).

Long-term tissue damage and abnormal inflammation have been shown as the main causes of post-COVID syndrome. Neuroinflammation, neurologic dysfunction, hypoxia, cerebral microvascular injury, metabolic aberrations in the brain after COVID-19 infection, endothelial dysfunction, latent viral reactivation, multi-organ pathology, and autonomic nervous system dysfunction have been thought to be other reasons for this syndrome (22).

Hopkins et al. (23) found that three out of five individuals continued to experience persistent fatigue, and more than one-fifth exhibited cognitive impairment 12 or more weeks after a confirmed COVID-19 diagnosis. Duggal et al. discovered that 33.2% of patients reported they were unable to fully recover after a COVID-19 infection, while Calabria et al. observed that 82.3% of 112 patients reported fatigue during the post-COVID period (24, 25). Ceban et al.’s (20) meta-analysis identified fatigue in 32% of patients and cognitive symptoms in 22%. Astin et al. (26) reported that myalgia, headache, and gastrointestinal symptoms were more common in female patients, whereas fatigue, respiratory issues, and cognitive symptoms were more frequent in older patients. In the present study, 90% (*n* = 36) experienced fatigue, and 10% (*n* = 4) suffered from fibrofog.

Frequently reported factors associated with a greater incidence of post-COVID syndrome amongst component studies included female sex, older age, greater severity of acute illness, and preexisting comorbidities. Elevated C-reactive protein and D-dimer levels were other factors that have been shown to be risk factors for persistent symptoms (20). In the current study, most of the patients were females, the average age was 55.50 ± 14.92, and they did not have severe illness. Perhaps this difference may be explained by the patient population in this study consisting of outpatient clinic patients rather than hospitalized patients.

In studies conducted on this patient group, rehabilitation programs were found to be extremely useful, and it was underlined that aerobic exercises, resistance exercises, and pulmonary rehabilitation should be included in the program. It is recommended that patients be evaluated for whether they are suitable for exercise or not when taking them into a rehabilitation program. Adding cognitive behavioral therapy to treatment has shown positive effects on quality of life (27). Although analgesics and nonsteroidal anti-inflammatory drugs may be used pharmacologically for pain, there is no pharmacological alternative in post-COVID syndrome as in COVID-19 infection (28). For this reason, the demand for rehabilitation programs and complementary medicine practices such as MBO is increasing day by day. It has been suggested that MBO may be used for both acute infection and the treatment of post-COVID symptoms (21). Tirelli et al. (10) performed MBO in 100 patients diagnosed with post-COVID syndrome. They applied 10 sessions of 40–50 μg/mL MBO 2–3 times a week and determined a statistically significant improvement in the Fatigue Severity Scale in 67% of the patients. The current study found improvement in fatigue, anxiety, depression, and quality of life after 10 sessions of MOT and supported Tirelli et al.’s findings.

In He et al.’s (29) randomized controlled study, 73 hospitalized patients with post-COVID syndrome were randomized into conventional therapy (*n* = 38) and conventional therapy plus MBO groups (*n* = 35). They applied 20–50 ng/mL MBO once a day for the last 7 days during hospitalization. The patients were evaluated with symptom scores, 6-min walk distance, pulmonary functions, and laboratory tests. A 50% decrease in symptom score and an increase in pulmonary functions, walking distance, and better laboratory results were obtained after the therapy. The results of the current study support these findings, but larger randomized controlled trials in polyclinic patients are needed.

Soldatenko et al. (30) conducted a randomized study including 140 patients with post-COVID syndrome, who were assigned to either pharmacological treatment alone or pharmacological treatment combined with ozone therapy. The pharmacological regimen consisted of Brainmax a combination of trimethylhydrazinium propionate and ethylmethylhydroxypyridine succinate recommended by the Russian Ministry of Health. The ozone therapy protocol included 10 sessions of intravenous infusion of 200 mL of ozonated 0.9% sodium chloride solution, with ozone concentrations gradually increased from 2.0 mg/L to 3.0–4.0 mg/L. Pre- and post-treatment assessments demonstrated greater increases in Insulin-like Growth Factor 1 (IGF-1), Brain-Derived Neurotrophic Factor (BDNF), and Nerve Growth Factor (NGF) levels, as well as more pronounced improvements in mental status, in the ozone therapy group compared with the pharmacological treatment group. In this study, no formal cognitive assessment was performed; only self-reported brain fog was documented, and objective biomarkers such as IGF-1, BDNF and NGF.

MBO enhances release of oxygen to tissues and exerts an anti-inflammatory effect by modulating prostaglandins. Additionally, it promotes the release of interleukin-1, interleukin-8, interleukin-12, and tumor necrosis factor, which help neutralize proinflammatory cytokines. The treatment also boosts metabolism and the excretion of intermediate metabolic products that activate pain receptors. Following O3 therapy, the release of endorphins increases, blocking pain signals in the thalamus and cortex. Moreover, hydrogen peroxide stimulates the pain-relieving system (31). Additionally, medical ozone modulates the Nrf2 (nuclear factor-erythroid 2–related factor 2) pathway in a way that promotes controlled antioxidant and cytoprotective responses. Pecorelli et al. showed that exposure of endothelial cells to ozonated human serum activates Nrf2, leading to an upregulation of heme oxygenase-1 (HO-1), a key antioxidant and anti-inflammatory enzyme. This indicates that mild, controlled oxidative stimulation by ozone can trigger an adaptive response that strengthens cellular defenses (31). Similarly, Re et al. (32) demonstrated *in vivo* that ozone pre-conditioning may activate the Nrf2/EpRE (electrophile-responsive element) pathway, supporting the idea that low-dose ozone can prime tissues to better withstand subsequent oxidative stress. Together, these findings suggest that medical ozone, when properly dosed, may confer benefits by enhancing endogenous antioxidant systems through Nrf2-mediated hormetic mechanisms. The treatment has been thought to be effective in post-COVID syndrome with the help of these mechanisms.

The main limitation of the study is the small sample size. Lack of prospective studies and lack of a control group are other limitations of the study. Because of this reason, further prospective studies, including larger sample sizes and control groups, are needed. Additionally, the pathogenesis of post-COVID and the effect of vaccines on post-COVID syndrome are still unknown. Also, there is a lack of standard dosage and session information for MBO in the literature. Specific studies for dosage and session should be designed. The major strength of the study is to evaluate patients’ fatigue, depression, and anxiety scores, sleep quality, and quality of life. Considering that pandemics have been recurring throughout history, complementary treatment methods such as MBO will remain important in relieving the symptoms seen after viral infections. More comprehensive studies on the subject are needed.

## 6 Conclusion

In the current study, improvement in fatigue, anxiety, depression, quality of life, and quality of sleep in patients diagnosed with post-COVID syndrome after 10 sessions of MBO was detected. The treatment may be preferred after COVID-19 infection in patients with persistent symptoms. Further studies are needed.

## Data Availability

The raw data supporting the conclusions of this article will be made available by the authors, without undue reservation.
